# Imaging of Osteosarcoma: Presenting Findings, Metastatic Patterns, and Features Related to Prognosis

**DOI:** 10.3390/jcm13195710

**Published:** 2024-09-25

**Authors:** Amandine Crombé, Mario Simonetti, Alessandra Longhi, Olivier Hauger, David Fadli, Paolo Spinnato

**Affiliations:** 1 SARCOTARGET Team, Bordeaux Research Institute in Oncology (BRIC) INSERM U1312 & University of Bordeaux, F-33076 Bordeaux, France; amandine.crombe@chu-bordeaux.fr; 2 Department of Skeletal Radiology, Pellegrin University Hospital, F-33076 Bordeaux, France; 3 Department of Radiology, Institut Bergonié, F-33076 Bordeaux, France; 4 Diagnostic and Interventional Radiology, IRCCS Istituto Ortopedico Rizzoli, 40136 Bologna, Italy; mario.simonetti2@studio.unibo.it; 5 Osteoncology, Bone and Soft Tissue Sarcomas, and Innovative Therapies, IRCCS Istituto Ortopedico Rizzoli, 40136 Bologna, Italy; alessandra.longhi@ior.it

**Keywords:** sarcoma, osteosarcoma, magnetic resonance imaging, diagnostic imaging, computed tomography, positron emission tomography, prognosis, response to treatment

## Abstract

**Background:** Osteosarcomas are rare malignancies (<1% of all cancers) that produce an osteoid matrix. Osteosarcomas are the second most frequent type of primary bone tumor after multiple myeloma and the most prevalent primary bone tumor in children. The spectrum of imaging findings of these malignancies varies significantly, reflecting different histological subtypes. For instance, conventional osteosarcoma typically presents with a mixed radiological pattern (lytic and bone mineralization) or with a completely eburneous one; aggressive periosteal reactions such as sunburst, Codman triangle, and soft-tissue components are frequently displayed. On the other hand, telangiectatic osteosarcoma usually presents as a purely lytic lesion with multiple fluid–fluid levels on MRI fluid-sensitive sequences. Other typical and atypical radiological patterns of presentation in other subtypes of osteosarcomas are described in this review. In addition to the characteristics associated with osteosarcoma subtyping, this review article also focuses on imaging features that have been associated with patient outcomes, namely response to chemotherapy and event-free and overall survivals. This includes simple semantic radiological features (such as tumor dimensions, anatomical location with difficulty of radical surgery, occurrence of pathological fractures, and presence of distant metastases), but also quantitative imaging parameters from diffusion-weighted imaging, dynamic contrast-enhanced MRI, and 18F-FDG positron emission tomography and radiomics approaches. Other particular features are described in the text. Overall, this comprehensive literature review aims to be a practical tool for oncologists, pathologists, surgeons, and radiologists involved in these patients’ care.

## 1. Introduction

Osteosarcomas are malignant mesenchymal tumors that develop from bone-forming sarcomatous cells and are the most common type of primary bone cancer—apart from those originating in the bone marrow [1,2]. Although considered rare, osteosarcomas account for about 20% of all primary bone tumors, making them the second most common malignant bone tumor after multiple myeloma. Osteosarcomas can be categorized as primary or secondary, with secondary osteosarcomas arising from a pre-existing condition or as a consequence of prior treatments, such as radiotherapy. The World Health Organization (WHO) classifies primary osteosarcomas into different subtypes, namely conventional (osteoblastic, fibroblastic, and chondroblastic), telangiectatic, low-grade central, small cell, parosteal, periosteal, and high-grade surface osteosarcomas [3,4]. Otherwise, secondary osteosarcomas represent malignant degeneration of benign entities such as Paget disease, large bone infarction, or post-radiotherapy. Osteosarcomas are also favored by germline abnormalities, such as Li–Fraumeni syndrome, Werner syndrome, or hereditary retinoblastoma [5].

These malignancies tend to show a dual peak of incidence: one in children, adolescents, and young adults and one in elderly patients (which, in this case, are more frequently secondary osteosarcoma and arise from plate bone). An older age has previously been associated with lower overall survival (OS) and event-free survival (EFS) [6,7].

Primary osteosarcomas typically prefer the meta-diaphysis of the long bones as the site of occurrence. In particular, the distal femur and proximal tibial represent about 50–65% of all sites. However, proximal locations in the femur and axial skeletons remain possible and have been linked to lower EFS, OS, and survival after metastasectomy [6,7,8].

A rare form of osteosarcomas is extraskeletal osteosarcoma (ESOS), which represents about 1% of all soft-tissue sarcomas and does not demonstrate any connection to the skeleton. It has the ability to form cartilaginous and osteoid matrices. Histologically, ESOS is completely indistinguishable from the classic osteogenic type. ESOS tends to present around the fifth to sixth decades of life, with a minor prevalence in men. It most frequently localizes to the lower limb in almost 50% of patients [9].

Further epidemiological characteristics depending on the osteosarcoma subtype are summarized in Table 1.

The diagnosis of osteosarcoma is confirmed through a pathological examination of biopsies, which must be properly sampled by an interventional radiologist, an oncologic radiologist with expertise in image-guided biopsy, or a surgeon from a bone sarcoma reference center. The biopsy should be performed using a co-axial technique, and the biopsy tract must be clearly marked to ensure its removal during curative surgery. Currently, curative management for patients with high-grade osteosarcoma includes perioperative chemotherapy (with doxorubicin, cisplatin, and methotrexate as the most common regimen), which has significantly improved the disease-free survival and R0 surgery [8]. A good response to neoadjuvant chemotherapy (defined as a necrosis rate > 90% of surgical specimen) is a strong but insufficient predictor of EFS and OS [10]. Afterwards, adjuvant chemotherapy is indicated [8].

In this comprehensive review, we aim to provide an overview of the role of imaging in the assessment of patients with osteosarcoma through the assessment of the imaging features associated with the diagnosis of osteosarcoma and its subtypes, the response to treatments, and, subsequently, EFS and OS.

## 2. Initial Radiological Features Associated with the Diagnosis of Osteosarcoma and Its Histological Subtypes

### 2.1. Imaging Modalities Recommended for Local, Regional, and Distant Staging in Osteosarcomas

Investigations should always start with conventional radiographs including two orthogonal planes and the entire bone.

A CT-scan is not mandatory but can be helpful to better examine the tumor matrix, notably calcification or bone formation, and the periosteal reaction. Hence, contrast-medium injection is generally not required.

If a malignancy cannot absolutely be excluded, radiographs must be complemented with contrast-enhanced MRI without delay. Indeed, the time to the final diagnosis and start of treatment should be as short as possible as it has been linked to patient outcome [11]. The MRI protocol should comprise at least T1-weighted imaging (WI) and T2-WI with and without fat suppression, followed by contrast-enhanced T1-WI with a slice thickness between 3 and 5 mm and in a plane resolution <1 × 1 mm^2^. The sequences must be acquired in at least two orthogonal planes. All of these sequences can be acquired using the DIXON method, which, notably, enables an analysis of the fat replacement due to the tumor on the fat phase, the peritumoral edema on the water phase of the T2-WI, and the hemorrhagic components on the water phase of the T1-WI in addition to the true T1 and true T2 signals of the bone tumor [12]. It is crucial to include at least one acquisition with a large field of view covering the whole bone in order not to miss a ‘skip metastasis’, i.e., a distinct bone metastasis in the remaining of the involved bone. It must be noted that baseline quantitative and functional MRI sequences, such as diffusion-weighted imaging (DWI) and dynamic-contrast enhanced MRI (DCE-MRI), can be helpful to evaluate the response to neoadjuvant treatments if a high-grade osteosarcoma is confirmed [13]. Interestingly, pseudo-CT MRI sequences with very short echo times can be useful to depict mineralization, osteolysis, the periosteal reaction, and destructions, but they are still to be evaluated in this context [14].

These examinations (X-rays ± CT scan and contrast-enhanced MRI) must be available to guide the biopsy.

Regarding the assessment of metastatic spreading, different options can be discussed depending on their availability [8]. The spread of the disease can at least be evaluated with chest CT and bone scintigraphy. However, it must be noted that (i) whole-body 18-fluoro 2-deoxy-D-glucose (18F-FDG) positron emission tomography (PET) CT, possibly combined with iodinated contrast-medium injection, and (ii) whole-body MRI are increasingly used for distant staging [8].

### 2.2. Radiological Findings on Conventional Radiographs

In both imaging and histology, conventional and secondary osteosarcomas are indistinguishable. They appear as aggressive, destructive bone masses with cloudlike bone formation and permeative osteolysis in long bones, which are classified as types II and III according to the Lodwick classification [15], and as a mass arising from a pre-existing condition, respectively [2].

**Figure 1:**
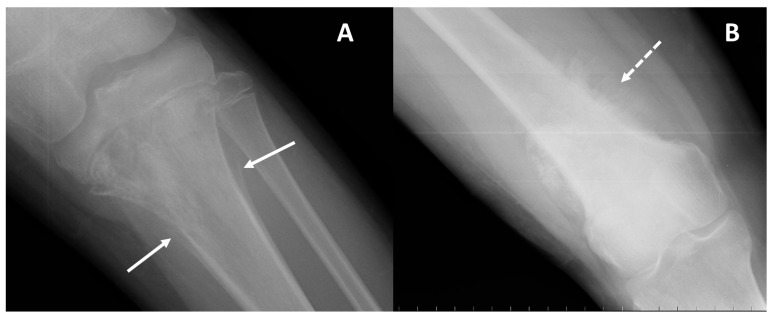
Periosteal reactions encountered on radiographs of osteosarcoma. (**A**) Conventional radiography of the left knee (AP projection) in an 11-year-old male with osteosarcoma of the proximal tibia presenting as a mixed (mainly lytic) pattern with an aggressive periosteal reaction: the ‘Codman triangle’ type (arrows). (**B**) Conventional radiography of the right thigh in a 55-year-old female with osteosarcoma of the distal femur presenting as a mixed (mainly eburneus) pattern with an aggressive periosteal reaction: the ‘Sunburst’ type (dotted arrow).

On conventional radiographs, i.e., first-line imaging, high-grade conventional osteosarcomas manifest as permeative lytic or mixed lesions with cortical erosion; periosteal reactions such as Codman triangle (Figure 1), sunburst, or hair-on-end patterns; and no sclerotic margins (on the contrary, blurry and irregular), which reflect their aggressive malignant nature [5,15]. It must be noted that small cell osteosarcoma, a very rare subtype of conventional high-grade osteosarcomas, lacks distinctive radiological features.

The other histological subtypes can demonstrate different radiological appearances that could guide diagnosis.

**Figure 2:**
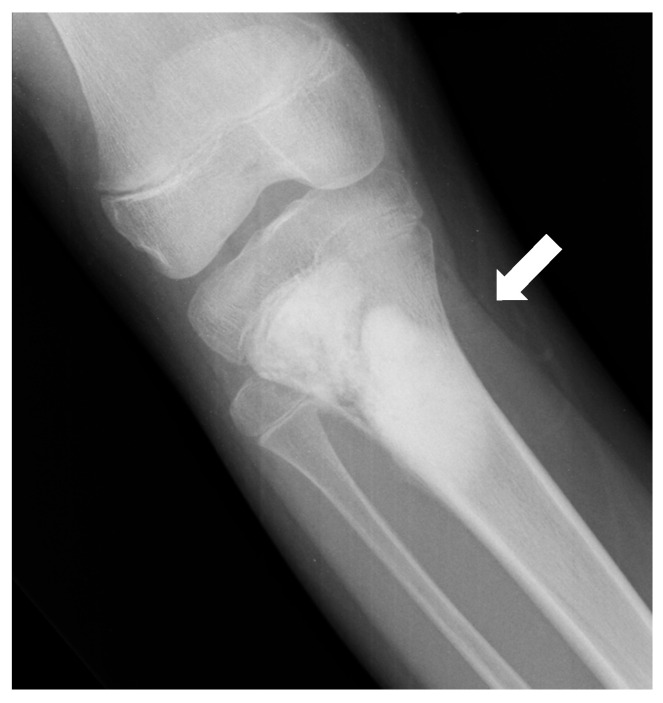
Pure bone-forming osteosarcoma. Conventional radiograph of the right knee (AP projection) of a 10-year-old female diagnosed with conventional (osteoblastic) osteosarcoma of the proximal tibia. A purely sclerotic pattern is observed at the proximal metaphysis of the tibia (arrow).

For instance, osteoblastic osteosarcoma and low-grade central osteosarcoma can present as a purely sclerotic pattern (Figure 2) and can be misdiagnosed as giant osteoma and enostosis [2,4,15,16,17,18,19,20]. Occasionally, it can be misdiagnosed with fibrous dysplasia on X-rays, as these bone tumors can also display both lytic and sclerotic areas in addition to a ground glass appearance.

**Figure 3:**
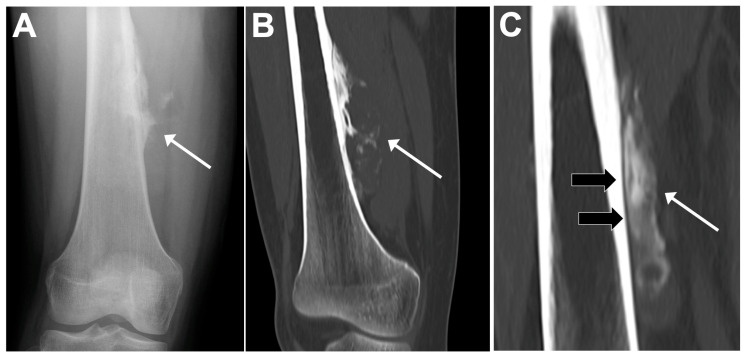
Parosteal osteosarcoma of the distal diaphysis of the femoral bone of a 20-year-old woman. (**A**) Conventional radiograph (AP projection) showing a bone-forming tumor at the surface of the diaphysis (white arrows). A CT scan can be useful to better depict the relationship to the cortex and periosteum (**B**,**C**). In particular, a thin radiolucent cleavage line separates the tumor from the bone cortex (black arrows).

Parosteal osteosarcomas arise from the outer layer of the periosteum, in general at the posterior face of the metaphysis of the distal femoral bone. They present as rather well-defined lobulated and exophytic masses with dense central mineralization and a thin radiolucent line separating them from the normally appearing cortex (Figure 3). Classical differential diagnoses include surface cartilaginous tumors, notably periosteal chondrosarcoma. Parosteal osteosarcomas are considered to have a good prognosis (5-year OS of about 85–90%), though they can transform to high-grade osteosarcoma with poorer OS.

**Figure 4:**
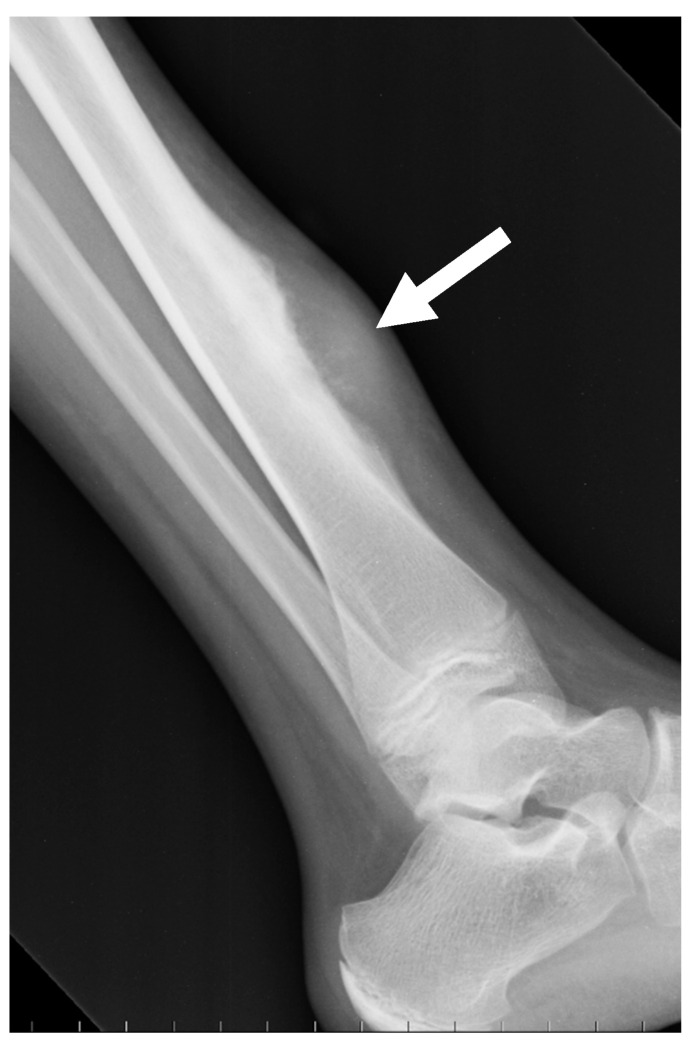
Periosteal osteosarcoma. Conventional radiography of the ankle (lateral projection) of a 13-year-old female with periosteal osteosarcoma of the distal tibia (meta-diaphyseal region); a disorganized aggressive periosteal reaction with partial superficial cortical disruption is seen on the anterior aspect of the tibia (arrow).

Periosteal ostesarcomas are other surface osteosarcomas arising from the inner germinative layer of the periosteum. They are considered intermediate-grade OS. They are much more lytic than parosteal osteosarcomas and combine irregular and spiculated cortical involvement with a heterogeneous mixed mass spreading in the soft tissue (Figure 4). Subtle lucency can be observed in the cortex and medulla. The periosteal reaction is often perpendicular to the cortex.

The last surface osteosarcoma corresponds to high-grade surface osteosarcomas, which resemble periosteal osteosarcomas but with a more aggressive presentation due to circumferential bone involvement, a larger size of up to 22 cm, and more extensive periosteal reactions and bone destruction.

Telangiectatic osteosarcomas are an atypical variant of osteosarcoma that can mimic primary and secondary aneurysmal bone cysts (for instance, secondary to giant cell tumors). This is due to the large hemorrhagic and necrotic cystic areas that make up almost the entire mass [2]. Asymmetric expansion, bone lysis, and an aggressive growth rate with limited periosteal reaction and poor bone component are other usual radiographic presentations of telangiectatic osteosarcomas.

**Figure 5:**
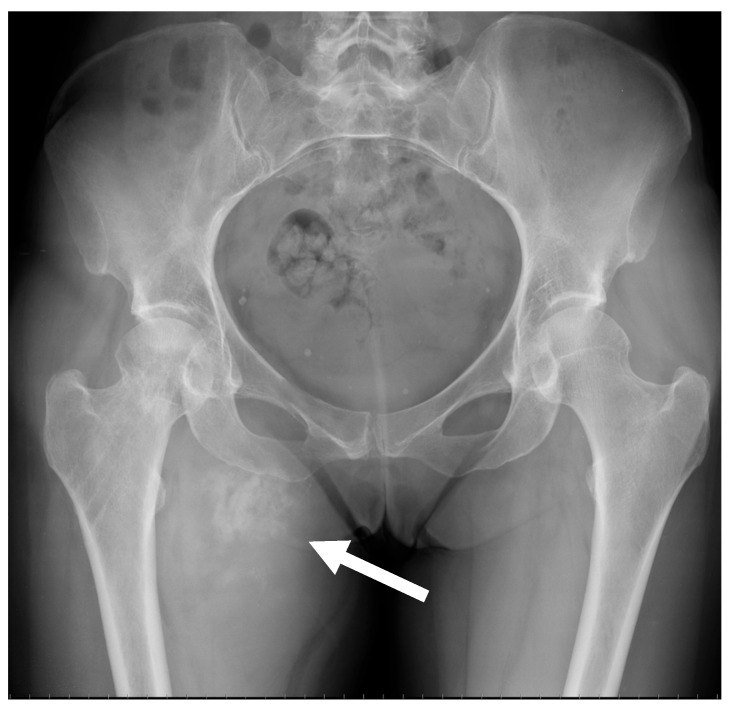
Conventional radiograph of the pelvis of a 51-year-old female diagnosed with extraskeletal osteosarcoma of the right proximal thigh in the abductor region. A mineralized mass with irregular large calcifications/ossifications can be observed (arrow).

ESOS frequently show up on radiographs as soft-tissue opacity with varying degrees of mineralization, which develop gradually with time (Figure 5) [9].

Radiographic investigation has limitations in precisely depicting the tumor matrix (i.e., chondroid, osteoid, hemorrhagic, fibrous, or cystic), the medullary involvement, and spreading in soft tissue; thus, for specific anatomical regions, such as the spine, iliac bones, or posterior parts of the vertebrae, where tissues overlap on 2D planes, traditional radiography is inadequate.

### 2.3. Radiological Findings on CT

A CT-scan is required as a complementary investigation to conventional radiographs. When radiographs are insufficient due to limited contrast resolution, multidetector CT enables detailed anatomical delineation and evaluation of the lesions in complex anatomical locations [21]. In addition, a CT-scan can show small calcifications, tumor mineralization, cortical alterations, and periosteal responses with greater accuracy than plain X-rays. A CT-scan allows to better appreciate the proportion of mineralized matrix compared to X-ray and MRI.

### 2.4. Radiological Findings on MRI

In MRI, the classic appearance of osteosarcoma is a mass with inhomogeneous low signal intensity on T1-WI sequence, high signal intensity on T2-WI, and intense enhancement after gadolinium chelate injection. However, bone-forming areas can display a low signal intensity on all sequences. The chondroid signal can be encountered in periosteal and parosteal osteosarcomas as well as dedifferentiated osteosarcomas. This signal is recognized as a low signal intensity on T1-WI, a very high signal intensity on T2-WI and heterogeneous, rather peripheral and lobulated contrast enhancement. Necrotic areas are characterized by a heterogeneous signal on T1-WI, possibly high after fat suppression due to bleeding, a high signal intensity on T2-WI, and no contrast enhancement.

It must be noted that fat-suppressed T2-WI can overestimate the real intramedullary extent of the tumor because of false positive findings due to peritumoral reactive marrow edema, and, in children, red marrow hyperplasia [22,23]. Hence, DIXON fat imaging and fat-suppressed contrast-enhanced T1-WI can be helpful to better depict the tumor margins.

**Figure 6:**
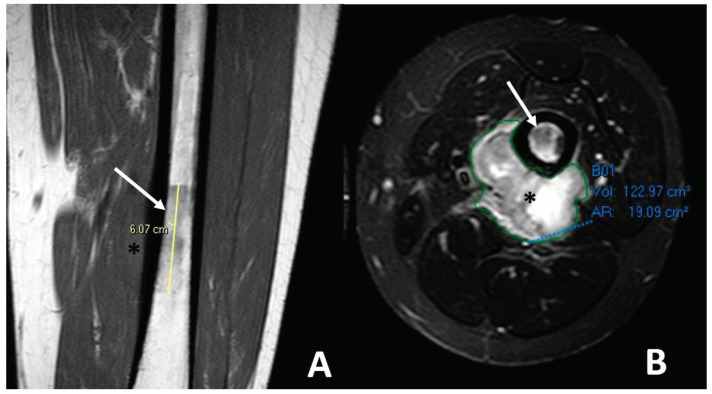
Soft tissue spreading in conventional high-grade osteosarcoma affecting the left femoral bone diaphysis in a 20-year-old male. (**A**) Coronal T1-weighted imaging (WI) showing the irregular intra-medullary involvement with a low signal intensity (white arrow) and a soft-tissue extension (black asterisk). (**B**) Axial T2-WI with fat suppression showing ill-defined soft-tissue spreading with high, heterogeneous signal intensities on T2-WI. Of note, the extra-osseous was automatically segmented (green line) for a further volumetric follow-up during neoadjuvant treatments.

MRI is also the best imaging modality to evaluate the extraskeletal soft-tissue component of osteosarcomas (Figure 6).

**Figure 7:**
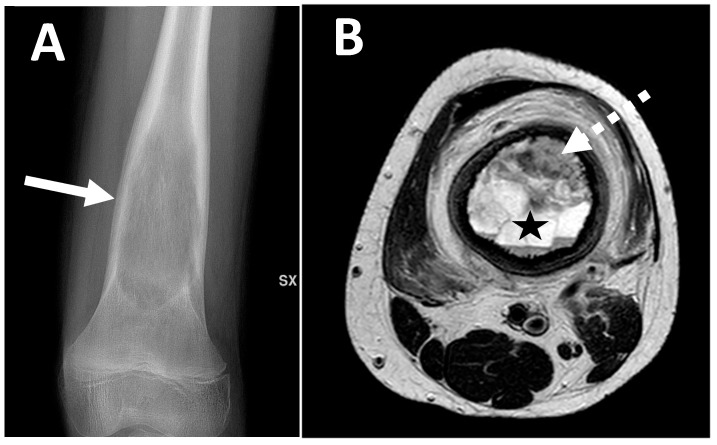
Conventional radiography of the left knee ((**A**) anteroposterior view) of a 13-year-old male with telangiectatic osteosarcoma of the distal femur metaphysis. A central expansile lytic lesion with limited multilayered periosteal reaction and sharply ill-defined margins can be observed (arrow). MRI ((**B**) axial T2-WI) shows multiple fluid–fluid levels with septa (star) and a concomitant solid area within the anterior aspect of the lesion (dotted arrow).

A typical MRI characteristic observed in telangiectatic osteosarcomas is the presence of fluid–fluid levels (Figure 7). MRI can also detect thick and irregular septa predominantly arranged in the edge and a solid enhancing component [24,25]. These septa represent the vital component of the lesion, so they may have a contextual bone matrix and will show vivid enhancement after contrast administration (Figure 7).

**Figure 8:**
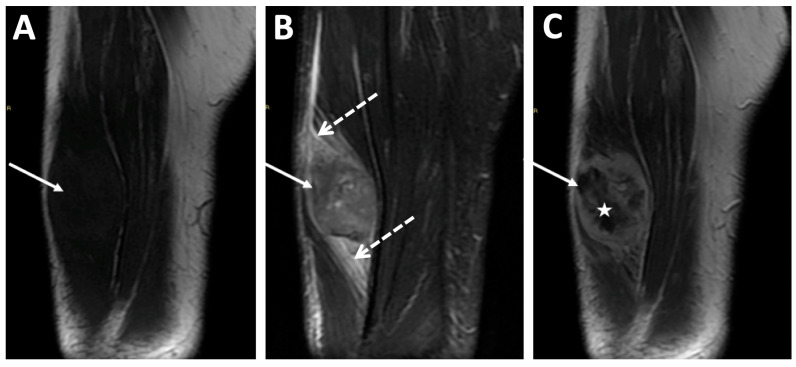
MRI of a high-grade extraskeletal osteosarcoma of the right thigh diagnosed in an 83-year-old male. (**A**) Coronal T1-weighted imaging (WI), (**B**) fat-suppressed coronal T2-WI, and (**C**) coronal T1-WI with gadolinium chelate injection. The tumor was seated deep in the anterior compartment of the thigh (arrows) and demonstrated a peritumoral edema (dashed arrows above and below the tumor) and inhomogeneous enhancement with internal necrosis (star).

Regarding extraskeletal osteosarcoma, both CT and MRI highlight well the complete discontinuity with neighboring bone segments. Contrast-enhanced T1-WI can demonstrate inhomogeneous enhancement due to the presence of areas of necrosis and mineralization together with a peripheral ‘rim’ enhancement (Figure 8) [9]. A classical differential diagnosis of extraskeletal osteosarcoma is myositis ossificans. To distinguish them, it can be useful to assess the presence of mature bone matrix with a predominantly peripheral arrangement, which is more frequently encountered in ossificans myositis and not osteosarcoma—though the specificity and sensitivity of this sign have not been evaluated. Other potential differential diagnoses include post-traumatic sequelae of bone avulsions at the tendon enthesis.

A summary of the wide spectrum of imaging features of different osteosarcoma subtypes at diagnosis is provided in Table 2.

## 3. Metastatic Patterns of Osteosarcoma

Osteosarcomas have a high tendency to metastasize. The hematogenous route is the most common spreading, and the lung is the most frequently affected organ. Indeed, about 10–20% of subjects affected by osteosarcoma present with synchronous metastases at the initial stage [26]. The most frequent metastatic site is the lung (80%), followed by the bones (30–35%) and lymph nodes (2%). If a metastatic disease is present at diagnosis, the prognosis of patients dramatically decreases from 60–70% to 10–30% in 5 years [26]. In addition, metachronous lung metastases occur in 40–55% of patients.

Thus, it is recommended to perform a thorax, abdomen, and pelvic contrast-enhanced CT-scan for distant staging and, after treatments, to detect distant recurrence. The place of 18F-FDG PET/CT is still controversial, but it appears more efficient to diagnose bone metastases compared to CT-scan alone [27]. Hence, the pooled sensitivity of PET/CT for detecting bone metastases in a meta-analysis of six studies was 93% with a pooled specificity of 97% [28].

Whole-body MRI (WB-MRI) could be an interesting alternative to detect metastatic spreading [29]. In a retrospective study of 36 patients with osteosarcoma, WB-MRI showed a sensitivity of 100%, a specificity of 96.3%, an accuracy of 97.3%, a negative predicted value (NPV) of 100%, and a positive predicted value (PPV) of 90.9% [30]. Moreover, in a prospective cohort of 54 patients with both Ewing sarcoma and osteosarcoma, Aryal et al. showed no significant difference in terms of diagnostic performances for detecting bone metastases between WB-MRI, 18F-FDG PET/CT, and 99mTc-MDP skeletal scintigraphy [31]. The optimization of the WB-MRI protocol with 3D Turbo Spin Echo DIXON sequences, high-quality DWI, and deep learning could enhance those diagnostic performances [32].

**Figure 9:**
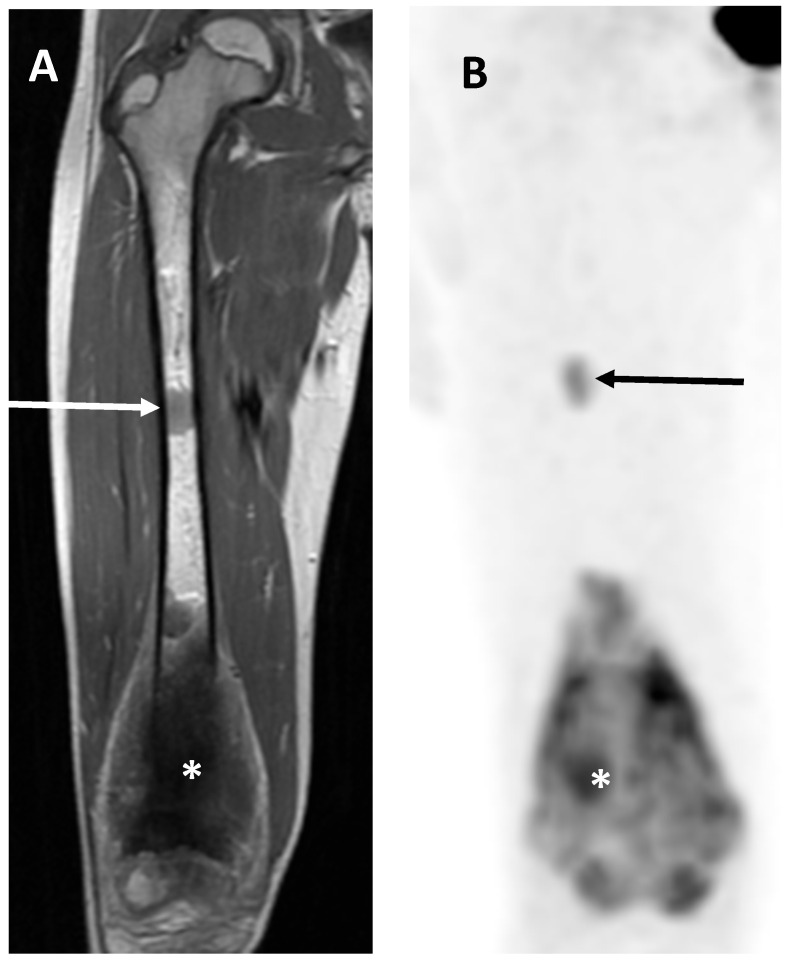
Skip metastasis. Coronal T1-weighted imaging (WI) (**A**) and view of 18F-FDG PET/CT (**B**) of 9-year-old female showing principal osteosarcoma mass of right distal femur (asterisks) together with proximal ‘skip metastasis’ (arrows).

Regarding skip metastases (Figure 9), Saifuddin et al. demonstrated that their prevalence was about 16%. Skip metastases were significantly associated with the presence of lung metastases (OR = 4.81, *p* < 0.001), other skeletal metastases (OR = 8.09, *p* < 0.001), and lower survivals (5-year OS = 45% with skip metastases versus 67% without; *p* < 0.001), but not with the response to chemotherapy in a large cohort of 241 patients with appendicular osteosarcomas [33]. It is still debated whether these skip lesions are primary synchronous localizations of disease due to spread either by contiguity or due to systemic spread. Overall, their reported prevalences range from 1% to 25%, which is likely due to variations in the methods used for detection (imaging versus histopathologic analysis) [34].

Multifocal osteosarcomas, or multicentric osteosarcomas, refer to osteosarcomas diagnosed with many bone metastases without lung metastasis. This can be considered as the occurrence of multiple synchronous bone metastases. In such cases, the radiologic pattern is almost constantly completely eburneous [35].

Lung involvement occurs by the hematogenous spread of tumor cells, which may manifest first as micronodules and then form coarse solid masses. According to Marcove et al., pulmonary metastases are almost always the first site of repetitive locations [36]. Differentiating between benign and malignant lung nodules in individuals with osteosarcoma can be difficult in cases of small nodule sizes [37,38,39].

**Figure 10:**
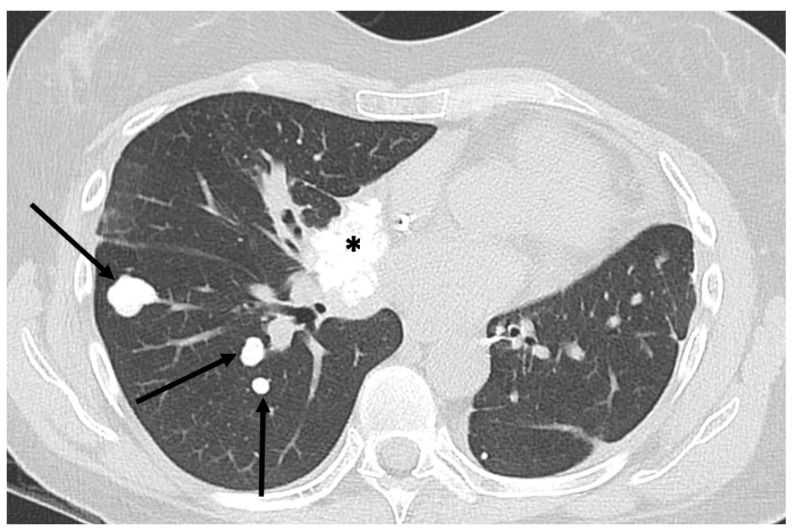
Mineralized bone metastases from extraskeletal osteosarcoma. Axial CT scan (lung kernel) in 54-year-old woman with calcified pulmonary (arrows) and mediastinal (asterisk) metastases.

Pulmonary metastases from osteosarcoma do not have pathognomonic features to distinguish them from other non-neoplastic diseases such as intrapulmonary lymph nodes, calcified sequela nodules, or chronic inflammation. Therefore, the most important parameter to consider is the size of the nodule; if it is >5 mm, it should be considered suspicious for malignancy, as demonstrated by the studies by Brader et al. [37] and Ghosh et al. [38]. In about 60% of cases, osteosarcoma lung metastases are partially or completely calcified, which can also be helpful (Figure 10) [38].

As shown by Bacci et al. [40], the number of lung metastases seems to correlate with the rapidity of onset. Indeed, early lung metastases tend to be found in greater numbers than late lung metastases, which are generally found in lower numbers.

The main metastatic patterns of osteosarcomas are summarized in Table 3.

## 4. Associations between Imaging Features and Patient Outcomes

As previously explained, therapeutic management in high-grade osteosarcomas relies on neoadjuvant chemotherapy followed by curative surgery. Several studies have demonstrated that a good response to neoadjuvant chemotherapy, i.e., >90% necrosis on a surgical specimen according to Huvos et al., is a strong independent predictor of EFS and OS as well as the quality of the surgical resection [6]. Consequently, it is crucial for imaging to distinguish good and poor responders as early as possible in order to adapt the systemic treatments. Three types of predictive features or imaging biomarkers extracted from medical images will be detailed in order of simplicity and development: (i) semantic radiological features, (ii) numerical features from quantitative and functional imaging, and (iii) radiomics features and models (Table 4).

### 4.1. Associations with the Response to Neoadjuvant Chemotherapy

Clinical studies have identified patient characteristics linked to the response to chemotherapy. Recently, in a retrospective cohort of 1702 patients, Bielack et al. showed that lower response rates were observed in male patients, axial tumors, and in patients with a long history of symptoms [6]. However, MRI is the best imaging modality to assess the initial tumor and monitor its changes during treatments [8].

#### 4.1.1. Semantic Radiological Findings

Semantic radiological findings refer to characteristics that are captured and explainable by radiologists.

**Figure 11:**
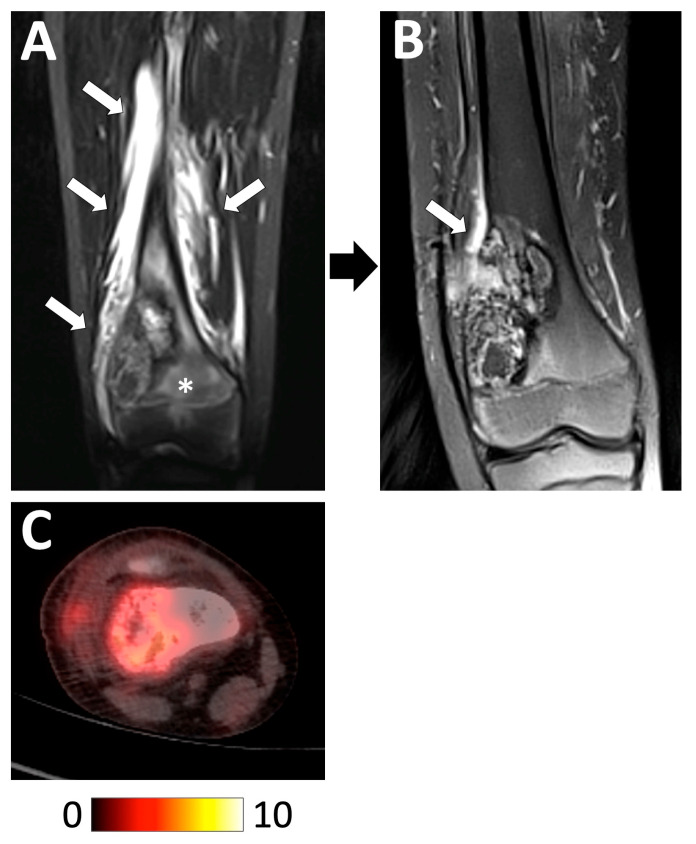
Examples of radiological features associated with a good histologic response. A 13 year-old boy diagnosed with a high-grade osteoblastic osteosarcoma of the distal right femoral bone was treated with perioperative chemotherapy. (**A**) Initial coronal STIR T2-weighted imaging showed the tumor with intramedullary edema (white asterisk) and extensive peritumoral edema in the surrounding tissues (white arrows). (**B**) At the end of chemotherapy, the tumor contours were better defined with a marked decrease in the intramedullary and soft-tissue edema. (**C**) Initial 18F-FDG PET/CT showing a moderately hypermetabolic tumor (baseline SUV_max_ = 5.65). The pathological analysis of the curative surgical resection revealed a good response (necrosis rate = 94.5%) and R0 margins. The patient is still alive without relapse 8 years later.

In a retrospective cohort of 57 patients, Holscher et al. showed that an increase in the tumor volume or no decrease in the amount of peritumoral edema between the baseline MRI and post-chemotherapy MRI could help identify poor responders. However, no odds ratio or multivariable analyses were performed in this study from 1992 (Figure 11) [41].

**Figure 12:**
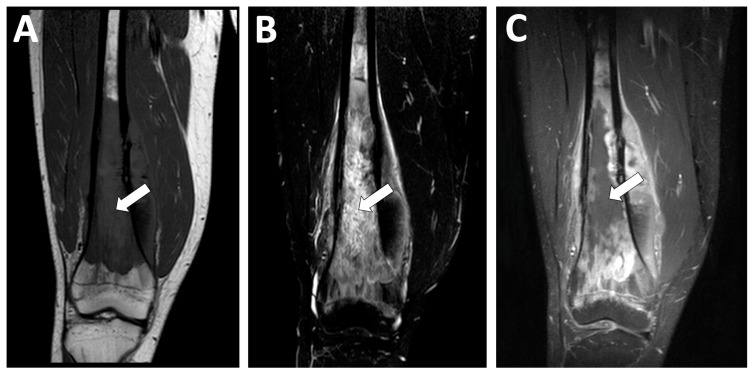
Examples of radiological features associated with a poor histologic response. A 15 year-old male presented with a high-grade osteoblastic osteosarcoma of the distal femoral bone. This tumor was characterized by a longest diameter of 16 cm, a volume > 150 mL, as well as central necrosis > 50% of the tumor volume (white arrows) with an intermediate signal intensity on (**A**) coronal T1-weighted imaging (WI), a high signal intensity in STIR (**B**), and no contrast enhancement on (**C**) fat-suppressed contrast-enhanced T1-WI. After completing neoadjuvant chemotherapy, a pathological analysis of the surgical specimen demonstrated a poor response (necrosis rate = 80%) and R0 margins. However, the patient is still alive without relapse 10 years later.

Recently, Kanthawang et al. evaluated another retrospective cohort of 95 patients with newly diagnosed high-grade osteosarcoma who underwent pre-treatment conventional MRI and had a post-chemotherapy histologic response. They found that a high tumor volume > 150 mL, a longest diameter > 7 cm, a necrotic area > 50% of the tumor volume, intra-articular spreading, and peritumoral soft-tissue edema were associated with a poor response in a univariable analysis (Figure 12) [42]. Additionally, the initial longest diameter and tumor volume were confirmed as independent predictors of the histologic response in multivariable analysis [42].

Interestingly, it seems that skip metastases at diagnosis are not associated with the response to chemotherapy [33].

#### 4.1.2. Quantitative and Functional Imaging

This type of imaging mostly refers to DCE-MRI, DWI, and PET/CT. The metrics (and their changes during treatment) extracted from the tumoral and peritumoral areas have been investigated in several studies as biomarkers of the response to treatment.

Regarding DCE-MRI, the shape of the time–intensity curve, the area under the time–intensity curve, the wash-in rate, the influx volume transfer constant (K^trans^), the efflux rate constant (K^ep^), the relative extravascular extracellular space (Ve), and the relative vascular plasma space (Vp) are employed as estimators of the tumor neoangiogenesis and have been the most studied estimators [43]. Overall, in a cohort of 69 patients with newly diagnosed, non-metastatic, high-grade osteosarcoma, Guo et al. showed in a univariable analysis that the K^trans^ and Vp values and the change in K^ep^ between the inner and outer parts of the tumor (i.e., an estimator of intra-tumoral fibrotic–necrotic changes) at intermediate evaluations were significantly associated with the histologic response [44]. More recently, in a retrospective cohort of 34 patients, Hao et al. also observed that (i) the K^trans^ value measured inside the tumor after completing neoadjuvant chemotherapy was significantly lower in good responders, and (ii) the change in K^trans^ from baseline to pre-surgical MRI was significantly more important in good responders [45]. Furthermore, in a two-center cohort of 85 patients, Kalisvaart et al. demonstrated that the optimal technique to estimate the DCE-MRI parameters in terms of diagnostic performances and inter-observer reproducibility was a whole slab segmentation of the tumor (instead of a focal area). Secondly, they showed that the relative wash-in rate (rWIR, measured by dividing the maximum slope of contrast enhancement on the time–intensity curve from baseline imaging by the wash-in rate post-chemotherapy) could help discriminate good from poor histologic responders using a cut-off of 2.3. Hence, they defined good radiological responders as those with an rWIR ≥2.3. The accuracy and area under the ROC curve (AUROC) of this imaging biomarker were 0.85 and 0.93, respectively, in the training cohort (n = 55) and 0.80 and 0.80, respectively, in the validation cohort (n = 30) [45].

Regarding DWI, the apparent diffusion coefficient (ADC) is hypothesized to reflect the tumor cellularity (with low ADC values in highly cellular tumors) and necrosis (with higher ADC values). The ADC has been measured in focal areas, cross-sectional areas, or 3D volumes at baseline and at regular time points during chemotherapy until surgery. In a meta-analysis of 13 studies including a total of 303 patients in which DWI was evaluated to predict the histologic response, Yuan et al. showed that, although there was high heterogeneity in the ADC values, the mean ADC difference before and after neoadjuvant chemotherapy and the ADC ratio were both significantly higher in good responders compared to poor responders [13]. The ADC has also been evaluated in the peritumoral tissue by Hao et al. [45]. These authors found that the ADC value measured in the peritumoral area after chemotherapy and its changes from baseline to post-chemotherapy were significantly higher in good responders.

Regarding 18F-FDG PET/CT, this imaging modality combines metabolic and anatomical imaging by using a radioactive glucose analog, which accumulates in high-glucose-uptake cells like cancer cells and helps assess the metabolic activity of tumors. Several studies have evaluated 18F-FDG PET/CT at baseline and its changes during chemotherapy to assess the histologic response in osteosarcomas through various numeric metrics, such as the maximal standardized uptake value (SUV_max_), the average SUV (SUV_mean_), the SUV_peak_ (measured in the 1 cm^3^ surrounding the SUV_max_ voxel), the tumor-to-background ratio (TBR), the total lesion glycolysis (TLG), and the metabolic tumor volume (MTV), as well as the PET evaluation response criteria in solid tumor (PERCIST) [46,47]. The predictive value of the baseline 18F-FDG PET/CT metrics remains debated [48]; however, it can be noted that Palmerini et al. found significantly higher response rates in patients with a low SUV_max_ (<6) at baseline compared to patients with a high SUV_max_ (64% versus 20%, *p* = 0.05) [48]. Oh et al. recently published an exhaustive review of the various cut-offs for PET/CT metrics during treatments [27]. It emerged that a post-chemotherapy SUV_max_ < 2–3, a TBR > 0.46–0.60, and a decrease in the SUV_max_ between the baseline and post-chemotherapy 18F-FDG PET/CT ≥ 52–60% were associated with a good histologic response, which was defined as >90% necrosis on surgical specimens [47,48,49]. Moreover, Xu et al. showed that the PERCIST criteria were more sensitive for detecting a response than the classical RECIST v1.1 criteria based only on one-dimensional changes [50].

#### 4.1.3. Predictive Radiomics Models

Radiomics is a recent field of research that has been extensively utilized in oncologic imaging this past decade [51,52]. It involves extracting numerous numerical features (named radiomics features) in order to quantify the shapes, textures, and patterns of intra-tumoral heterogeneity from 3D segmentations of primary tumors, metastases, or their surrounding tissues. These thousands of radiomic features are generally analyzed using supervised machine learning algorithms and data science techniques to account for their collinearity and high dimensionality [53] in order to identify predictive signatures for diagnosing malignancy, staging and grading cancers, and predicting responses to treatment and survival [54,55].

Radiomics approaches to predict histologic responses in patients with osteosarcoma have been mostly applied in pre-treatment MRI (particularly T2-WI and contrast-enhanced ± fat-suppressed T1-WI) and CT-scan. Two studies have also employed deep learning algorithms to automate segmentation before utilizing the usual radiomics workflow [56,57]. Although their methods were retrospective, heterogeneous, and improvable (with an average radiomics quality score of 20.6% [6.92/36]), making it difficult to compare them [58], they all obtained strongly encouraging results in independent validation sets. Indeed, the AUROC for the best models in testing sets ranged between 0.71 and 0.97 [59,60]. Interestingly, the model performances were systematically improved when clinical data were added to radiomics data [56,57,58,59,60,61]. Similarly, post-chemotherapy radiomics data also enhanced the predictive performances of the baseline radiomics models [62].

Yet, it must be emphasized that all of these potential biomarkers of the histological response to neoadjuvant chemotherapy remain to be validated prospectively in multiple-center studies.

### 4.2. Associations with EFS and OS

**Figure 13:**
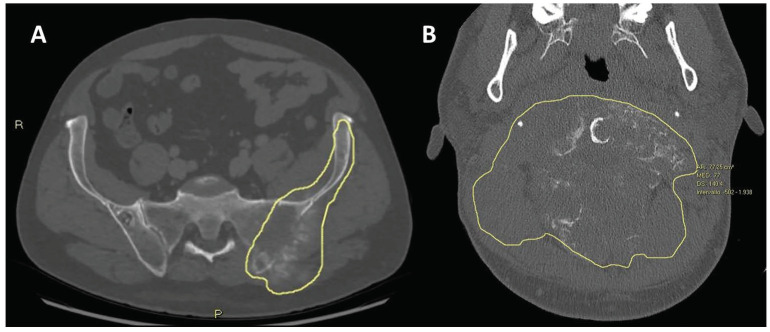
Baseline CT scans of two patients affected by osteosarcomas located in skeletal segments difficult to reach surgically, namely (**A**) iliac bone and (**B**) upper cervical spine. Tumor margins are depicted with yellow lines. Both lesions were declared inoperable by multidisciplinary tumor board from sarcoma reference center.

First, several clinical, biological, and histological features have been associated with patient survival. A lower EFS has been reported in the large EURAMOS-1 cohort that included more than 2000 patients with the following characteristics: an older age (HR = 1.25 and *p* = 0.013 for adolescents and HR = 1.32 and *p* = 0.008 for adults—with children as reference), male patients (HR = 1.20; *p* = 0.017), a proximal femur or humerus location (HR = 1.50; *p* < 0.001) and an axial location (HR = 1.53 and *p* = 0.011—with another limb site as reference; Figure 13), pulmonary metastases (HR = 2.34; *p* < 0.001) as well as other non-lung metastases (HR = 1.94; *p* < 0.001), the WHO subtype (with conventional non-osteoblastic non-chondroblastic, telangiectatic, and surface osteosarcomas demonstrating a better EFS compared to conventional chondroblastic osteosarcomas—HR = 0.67 and *p* = 0.003; HR = 0.52 and *p* = 0.003; and HR = 0.44 and *p* = 0.047, respectively), and a large tumor volume ≥ 1/3 of the involved bone (HR = 1.29; *p* = 0.002) [63]. It must be noted that larger tumor size and volume were associated with the OS and EFS in other studies using different categorizations [64,65].

More precisely, the presence of metastases at the diagnosis decreases the 5-year survival rate from about 70% to 20% [26]. Patients with a tumor ≥10 cm have a survival rate of 40% compared to 65% for other patients. There is a twofold increase in the risk of mortality during the first 5 years following diagnosis for individuals with larger tumors.

Logically, these features were subsequently associated with the OS in this cohort according to multivariable Cox regression, except for surface osteosarcoma (probably due to small effectives), adult patients compared to children, and a large tumor volume ≥1/3 of the involved bone—though similar trends were observed [66,67].

In addition, a poor histologic response and incomplete surgical resections have also been associated with poorer OS and EFS and have even been reported as key prognostic factors in a multivariable analysis of 1702 patients by Bielack et al. [6].

While the presence of a pathological fracture at diagnosis was not associated with the OS and EFS in multivariable modeling in the EURAMOS-1 cohort [7], opposite findings were observed in the adult subgroup of patients in another study of 2847 patients registered in the Consecutive Cooperative Osteosarcoma Study Group database [68]. Indeed, Kelley et al. observed a significantly lower OS in case of pathological fracture (HR = 1.89; *p* = 0.013). However, no significant results were seen for the EFS in adults and for the OS and EFS in children [68].

Lastly, initial lactate dehydrogenase (LDH) and alkaline phosphatase (ALP) have been inconstantly linked to patient outcomes, with lower LDH and lower ALP possibly being associated with better EFS [69,70].

#### 4.2.1. Semantic Radiological Findings

**Figure 14:**
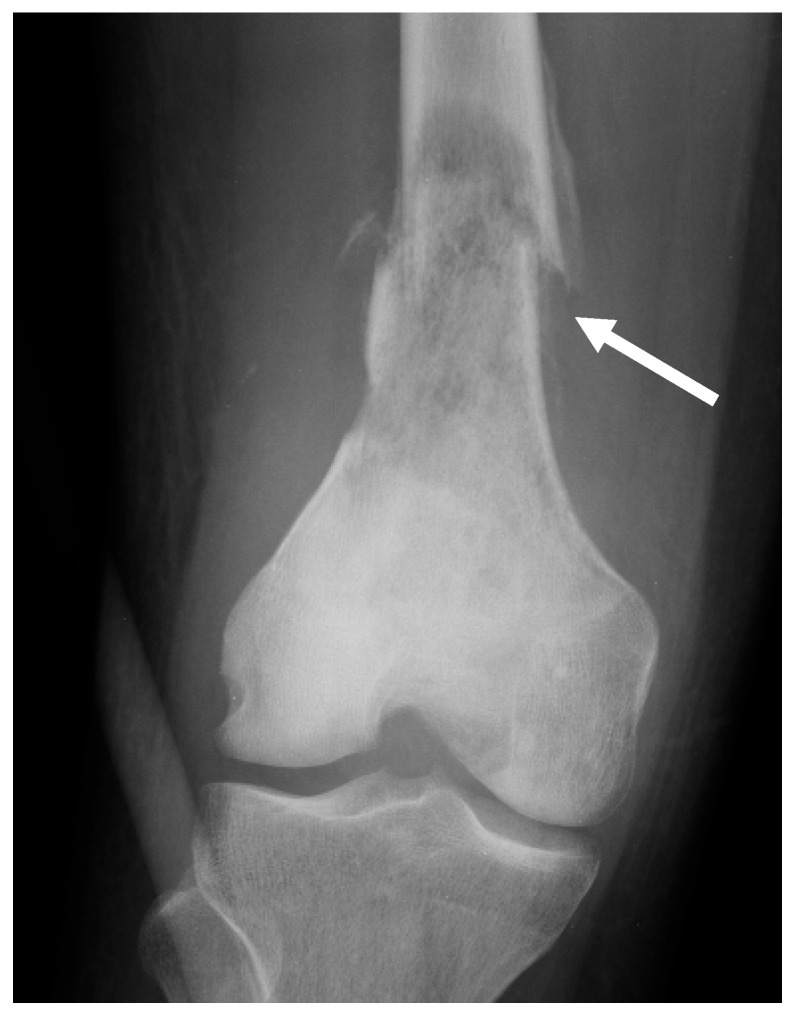
Pathological fracture on conventional radiography (AP view) of a 22-year-old male diagnosed with conventional (fibroblastic) osteosarcoma taken during neoadjuvant chemotherapy (arrow).

Though categorized as clinical characteristics in these surgical and medical studies, the tumor size and volume, the location, pathological fractures (Figure 14), and the presence and patterns of metastases are obtained through conventional imaging. Moreover, as seen above, several qualitative and quantitative imaging features are associated with the histological response, which is associated with the OS and EFS.

Other semantic features may help identify patients at risk of poorer OS and EFS.

Neurovascular involvements have been associated with an increased risk of local recurrence, which, in turn, results in a drop in the overall survival at 5 years to about 15% [17]. However, neurovascular involvements can be difficult to confirm in imaging when there is not a complete circumferential encasement or an intra-lumen tumor bud. Hence, the diagnostic performances of MRI to diagnose vascular involvement were a sensitivity of 100%, a specificity of 61.1%, a positive predictive value 53.3%, and a negative predictive value 100% [71].

Regarding transphyseal spreading in children, its prognostic impact seems unclear, but it precludes the preservation of the joint during curative surgery [71].

**Figure 15:**
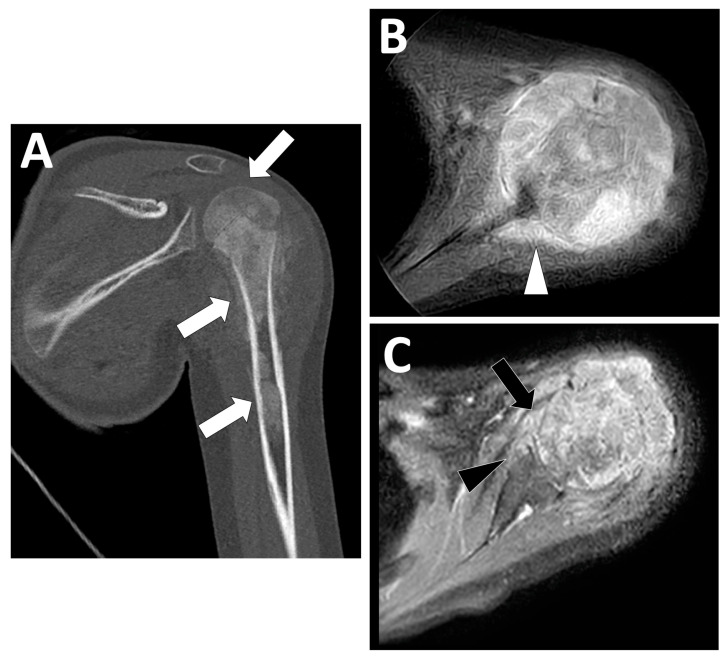
Transphyseal and intra-articular spreading observed during the initial staging of a high-grade osteoblastic osteosarcoma of the proximal left humeral bone diagnosed in a 12-year-old girl. (**A**) Coronal view of the initial CT scan showing an extensive tumor (>15 cm long) involving the epiphysis, metaphysis, and diaphysis of the humerus (white arrows). (**B**,**C**) Axial contrast-enhanced T1-weighted imaging showing marked contrast enhancement surrounding the scapula (white arrowhead) with periosteal enhancement, fulfilling of the articular recessus (black arrow) and erosions of the anterior side of the glenoid (black arrowhead).

Regarding intra-articular spreading, similarly, there is a lack of objective findings about its impact on patient survival. However, it will modify the surgical approach. In a recent study by Bodden et al., the following MRI features were significantly associated with joint invasion: direct visualization of the intra-synovial tumor tissue (OR = 186–229; *p* < 0.001) and the destruction of the intra-articular bone (OR = 69–324; *p* < 0.001). Furthermore, replacement of the epiphyseal bone marrow and contrast enhancement of the synovial were the most sensitive indirect signs (96%) but with limited specificities (29–54%) [72] (Figure 15).

In patients with extraskeletal osteosarcoma, the presence of large internal necrosis/and or internal hemorrhagic areas and signal intensity inhomogeneity on T2-WI sequences seem to correlate with poorer prognosis [9].

#### 4.2.2. Quantitative and Functional Imaging

Regarding DCE-MRI, some initial parameters and their changes during neoadjuvant chemotherapy seem to correlate with survival rates, though in small retrospective studies. Guo et al. also found that the difference between the outer and inner values of Ve at baseline was associated with the EFS (*p* = 0.002 with an optimal cut-off of 0.026). However, these findings were not confirmed in another center or prospectively [45]. Moreover, Kalisvaart et al. confirmed the ability of the rWIR with a cut-off of 2.3 to predict the EFS in a two-center cohort of 82 patients [13]. Indeed, rWIR < 2.3 (i.e., a poor radiological response) was associated with a lower EFS (HR = 2.4, 95%CI = 1.1–5.0 in the entire population; HR = 2.3, 95%CI = 1.0–5.2 in patients without metastasis at diagnosis) [73]. Lastly, Hao et al. observed that a lower baseline peritumoral V^e^ (with a cut-off of 0.2485) and lower post-chemotherapy intra-tumoral K^trans^ (with a cut-off of 0.2275) were associated with a longer EFS (*p* = 0.016 and *p* < 0.001, respectively) [13]. Moreover, the peritumoral V^e^ and intratumoral K^trans^, both at baseline and after chemotherapy, could help identify patients with a longer OS (*p*-value range: <0.001–0.023)—with lower values indicating longer survivals [13].

Regarding DWI and survival rates, Hao et al. also investigated this sequence in the same study of 34 patients and demonstrated an association between a higher post-chemotherapy ADC in the peritumoral tissue and a longer OS (*p* = 0.035) [45]. Yet, these results should be confirmed in a larger prospective and independent cohort in a multivariable setting.

Regarding 18F-FDG PET/CT and EFS or progression-free survival (PFS), high baseline SUV_max_ (cut-off: 15), baseline MTV (cut-off: 238), baseline TLG (cut-off 35.8), and post-chemotherapy SUV_max_ (cut-off: 2.5–5) and low TLG change (cut-off: 10%) were linked to worse outcomes in three retrospective studies comprising a total of 105 patients [27,74,75]. In terms of OS, higher baseline TLG (cut-off: 1022), baseline MTV (cut-off: 238), and post-chemotherapy SUV_max_ (cut-off: 3.3) seemed to correlate with lower OS [27,74,75]. In addition to 18F-FDG, other radiotracers have been explored in patients with osteosarcoma. Notably, 18F-NaF (sodium fluoride) PET/CT targets the skeleton by being directly absorbed onto the surface of the bone matrix, which can be used to detect bone metastases (with better performances than technetium-99m-labeled methylene diphosphonate bone scan) and extra-osseous mineralized metastases in various cancers, especially osteosarcomas [27]. However, the potential added value of 18F-NaF PET/CT to predict treatment responses, including the EFS, PFS, and OS, has not yet been elucidated (Table 5).

#### 4.2.3. Predictive Radiomics Models

Few proof-of-concept studies have investigated the ability of radiomics to predict the OS and EFS in osteosarcoma with a heterogeneous retrospective design (i.e., based on T2-WI, ADC map, or contrast-enhanced T1-WO) and outcomes (i.e., 1-year survival, OS, and DFS) and a lack of prospective external validation. Nonetheless, all studies found encouraging results with high concordance indices whatever the outcome to predict (c-index range: 0.741–0.813), and there was an increase in the performances of the clinical model when radiomics data were added to the modeling [60,76,77].

Table 4 and Table 5 summarize the main qualitative and quantitative imaging features, respectively, associated with prognosis in patients with osteosarcoma.

## 5. Conclusions

In this comprehensive review, we summarized the main radiological patterns of osteosarcomas at the initial diagnosis, their metastatic patterns, and the potential imaging biomarkers that could aid in predicting the response to neoadjuvant chemotherapy and in prognostication. This review was based on an exhaustive analysis of the literature involving conventional radiographs, CT scans, MRI, 18F-FDG PET/CT, and innovative radiomics approaches. All of these imaging modalities play crucial roles in the staging, monitoring, and follow-up of these malignant tumors. Imaging holds a pivotal role in the diagnostic and therapeutic management of osteosarcomas, with significant improvements anticipated through advancements in quantitative imaging, radiomics, and artificial intelligence. However, the rarity of osteosarcomas and the lack of multiple-center radiological databases led to a shortage of validated imaging biomarkers for patients with osteosarcoma. Moreover, studies combining these different imaging modalities with other clinical, biological, and molecular biomarkers are missing and would be helpful to better understand the correlations between imaging biomarkers and improve predictive modeling. Future prospective and collaborative initiatives among radiologists are anticipated to overcome these challenges and enhance patient outcomes.

## Figures and Tables

**Table 1:** The epidemiological features of the subtypes of osteosarcoma.

Osteosarcoma Subtype	Frequency	Age and Sex Prevalence	Skeletal Regions Most Frequently Involved
Conventional high-grade osteosarcoma ^1^	75–80%	10–20 years old; M:F = 1.5:1	Distal femoral bone, proximal tibia, or humerus (70%)
Telangiectatic ^1^	2–12%	10–20 years old; M:F = 1.5:1	Around knee or shoulder joint (70%)
Secondary ^1^	4%	50–80 years old; M:F = 1.5:1	Around knee or hip joint
High grade of bone surface ^1^	1–2%	10–40 years old; M:F = 1.5:1	Tibia and femur (both near knee and in diaphysis)
Small cell ^1^	1%	5–25 years old; M:F = 1:1.5	Around knee joint
Extraskeletal ^1,^*	2–5%	50–80 years old; M:F = 1:1.5	Thigh (soft tissue)
Parosteal ^2^	<4%	10–45 years old; M:F = 1:1.5	Around knee joint (in 60% of cases, posterior aspect of distal femur)
Central low-grade ^2^	2–3%	10–50 years old; M:F = 1:1	Around knee joint
Periosteal ^2^	>1%	10–20 years old; M:F = 1.5:1	Tibia and femur (both near knee and in diaphysis)

^1^ High grade, ^2^ low grade, * soft-tissue location.

**Table 2:** A summary of the main radiologic features of initial presentations of the different osteosarcoma subtypes.

Osteosarcoma Subtype	Radiologic Pattern	Periosteal Reaction	Other Imaging Features
Conventional osteosarcoma	Mixed (lytic and sclerotic) or completely eburneous	Sunburst, Codman triangle, other irregular/aggressive types	Soft-tissue components frequently displayed
Telangiectatic	Purely osteolytic	None or thin regular	Multicystic pattern with fluid–fluid levels and solid components on MRI
Secondary	Lytic heterogeneous	Aggressive types	Different depending on pre-existing lesions
High-grade of bone surface	Mixed mineralized and non-mineralized soft-tissue components without cleavage with bone cortex	Uncommon	Cortex erosion (50%), intramedullary involvement (50%)
Small cell	Usually similar to classic osteosarcoma	Usually similar to classic osteosarcoma	Usually similar to classic osteosarcoma
ESOS	Soft-tissue mass with inhomogeneous contrast enhancement, various degrees of internal necrosis	None	Internal mineralization frequently displayed (60%)
Parosteal	Lobulated osseous mass fused with cortical bone, usually with large dimensions, broad implant base	None, non-aggressive ones, or only cortical thickening	Cauliflower-like mass, thin linear cleavage between portions of tumor and cortical bone, frequent intramedullary involvement
Central low grade	Lytic, eburneous, or mixed, usually with large dimensions	If present, regular/non-aggressive	Soft-tissue mass (50%)
Periosteal	Periosteal dense mass with well-defined borders	None or sometimes Codman triangle	Cortex may be intact or focally eroded, but bone canal is not involved

**Table 3:** Metastatic patterns of osteosarcoma.

Metastases at Diagnosis	Lung Involvement	Bone Involvement
15–20%	80%	10–30%
	60% calcified or ossified	10–25% skip metastases, while locations in other bones are rarer
	40% non-calcified	

**Table 4:** A summary of the main qualitative features derived from imaging studies associated with a poor prognosis in patients with osteosarcoma.

Imaging Feature	Clinical Significance
- Large tumor size and volume (longest diameter > 7–10 cm, volume > 150 mL, volume >1/3 of involved bone) - Increased tumor volume and diameter during NACT	- Lower response rate to NACT - Decreased OS and EFS
- Intra-articular tumor spreading	- Difficult to perform limb-sparing surgery - Increased risk of local recurrence - Decreased OS
- Proximity to major blood vessels	- Difficult to perform limb-sparing surgery - Increased risk of local recurrence - Decreased OS
- Location in difficult sites for surgery (axial skeleton, girdles, skull base, proximal humerus, or femur)	- Impossibility of surgery or difficulty in removing whole tumor - Decreased OS and EFS
- Occurrence of pathological fracture	- Tumor spread outside of bone and/or hematic spread - Decreased OS and increased risk of local recurrence (debated)
- Presence of distant metastases	- Decreased OS
- MRI signal of internal hemorrhagic areas *	- Decreased OS
- MRI signal intensity inhomogeneity (T2-WI) *	- Decreased OS
- Necrotic area > 50% of tumor volume	- Lower response rate
- Peritumoral soft tissue edema - No decrease in peritumoral edema during NACT	- Lower response rate to NACT

* Extraskeletal osteosarcomas. Abbreviations: EFS: event-free survival, NACT: neoadjuvant chemotherapy, OS: overall survival, WI: weighted imaging.

**Table 5:** A summary of the main quantitative features derived from imaging studies associated with a poor prognosis in patients with osteosarcoma.

Modality, Sequence	Quantitative Imaging Data	Clinical Significance
DCE-MRI	Lower K^trans^ and V^p^, change in K^ep^ between inner and outer tumor areas at intermediate evaluation	Good histologic response
	Lower K^trans^ measured inside tumor after NACT	Good histologic response
	Decrease in K^trans^ from baseline to pre-surgical MRI	Good histologic response
	Relative wash-in rate ratio (rWIR) < 2.3 (poor radiological response)	Poor histologic response
	Difference between outer and inner values of V^e^ at baseline	Lower EFS
	Lower baseline peritumoral V^e^ (cut-off = 0.2485) and lower post-chemotherapy intra-tumoral K^trans^ (cut-off = 0.2275)	Longer EFS and OS
	Lower intra-tumoral K^trans^ at baseline and after chemotherapy	Longer OS
DWI	- Mean ADC difference before and after NACT - ADC ratio significantly higher in good responders compared to poor responders	Higher decrease in good histologic responders
	- Peritumoral ADC measured in peritumoral area after NACT - Changes in peritumoral ADC from baseline to end of NACT	Higher decrease in good histologic responders
	Higher post-chemotherapy peritumoral ADC	Longer OS
18F-FDG PET/CT	Low baseline SUV_max_ (cut-off = 6)	Good histologic response
	High baseline SUV_max_ (cut-off = 15)	Lower EFS and PFS
	Low post-chemotherapy SUV_max_ (cut-off = 2–3)	Good histologic response
	High post-chemotherapy SUV_max_ (cut-off = 2.5–5)	Lower EFS and PFS
	TBR > 0.46–0.60	Good histologic response
	Decrease in SUV_max_ between baseline and post-cNACT ≥ 52–60%	Good histologic response
	PERCIST criteria	Good histologic response
	High baseline MTV (cut-off = 238),	Lower EFS, PFS, and OS
	Higher baseline TLG (cut-off = 1022),	Lower OS
	Low TLG change (cut-off = 10%)	Lower EFS and PFS

Abbreviations: ADC: apparent diffusion coefficient, DCE-MRI: dynamic contrast-enhanced MRI, EFS: event-free survival, MTV: metabolic tumor volume, NACT: neoadjuvant chemotherapy, OS: overall survival, PFS: progression-free survival, SUV: standardized uptake value, TBR: tumor-to-background ratio, TLG: total lesion glycolysis.

## Data Availability

Additional data and further information can be obtained from the corresponding author.
